# Available Genetic Data Do Not Support Adaptation of *Tobacco Ringspot Virus* to an Arthropod Host

**DOI:** 10.1128/mBio.01875-16

**Published:** 2017-01-24

**Authors:** Robert S. Cornman

**Affiliations:** US Geological Survey, Fort Collins Science Center, Fort Collins, Colorado, USA; Virginia Tech; University of Nebraska

## LETTER

*Tobacco ringspot virus* (TRSV), a nepovirus, is a long-studied, economically important plant pathogen. Infectious particles are found in pollen and seed and are also vectored by arthropods and nematodes (1). Li and colleagues (2) presented direct evidence of the replicating strand of TRSV in honey bee tissues and showed that TRSV sequence detection rates correlated with season and hive strength in the same manner observed for other viruses known to infect bees. While additional confirmation is desirable (3,4), these observations remain compelling indications that TRSV infects and replicates in honey bees. In contrast to assertions elsewhere (5), detection of TRSV in metagenomic sequence data of honey bees (6) was never represented as evidence of infection.

**FIG 1:**
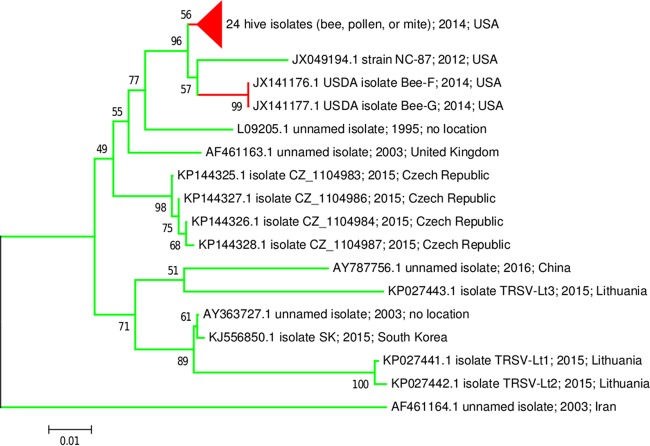
Unrooted phylogenetic tree derived from a 576-bp codon-aligned TRSV capsid region. The tree was constructed in MEGA6 (7) using the maximum composite likelihood mutational model and neighbor-joining tree reconstruction. Bootstrap support values are for 1,000 replicates. Plant-derived sequences are indicated in green, and hive-derived sequences are indicated in red. Labels include GenBank accession number, isolate name (if any), source country (if stated), and accession number release date. The tree and data matrix are available in TreeBase (http://purl.org/phylo/treebase/phylows/study/TB2:S19985).

On the other hand, Li and colleagues (2) also presented a phylogenetic argument suggesting that the TRSV lineage that they had identified from beehives was evolutionarily divergent from plant isolates. They postulated that this divergence was driven by adaptive evolution of TRSV to a new host from a different kingdom of life. A new phylogenetic analysis of all public TRSV sequences available in GenBank as of 1 September 2016 that adequately overlap the capsid locus analyzed in reference 2, following their methodology, did not support this conclusion (Fig. 1). The hive-derived sequences are in fact well within the spectrum of TRSV variants identified from plants. The phylogenetic pattern of reciprocal monophyly obtained by the analysis described in reference 2 was due to oversampling of essentially the same biological material (independent TRSV infections from geographically distinct areas were not available) and an incomplete representation of TRSV diversity. The new analysis showed that multiple sets of plant-derived TRSV have high bootstrap support and longer basal branch lengths than the set containing the bee-derived sequences, which are no longer monophyletic.

More telling than the new phylogenetic topology is the fact that no fixed nucleotide differences were observed in the alignment between plant-derived and hive-derived TRSV sequences (see character matrix in TreeBase; http://purl.org/phylo/treebase/phylows/study/TB2:S19985) from which Fig. 1 was derived. Thus, the lack of monophyly for bee-associated TRSV is not simply due to long-branch attraction between those sequences and another divergent TRSV lineage. Hive-associated sequences are neither novel nor outliers in the spectrum of sequence variation found in plant TRSV from multiple continents. The conclusion that TRSV has undergone divergent evolution to adapt to a novel host is no longer supported by the available genetic data.
